# Seronegative Morvan Syndrome Presenting With Agrypnia Excitata and Peripheral Nerve Hyperexcitability: A Phenotype‐Supported Diagnostic and Therapeutic Approach in a Resource‐Limited Setting

**DOI:** 10.1002/ccr3.73196

**Published:** 2026-07-22

**Authors:** Hatem Mousa Taha, Khaled Hatem Taha

**Affiliations:** ^1^ Faculty of Medicine and Health Sciences, An‐Najah National University Nablus Palestine; ^2^ Department of Internal Medicine Palestine Medical Complex Ramallah Palestine

**Keywords:** agrypnia excitata, immunotherapy, Morvan syndrome, neuromyotonia, peripheral nerve hyperexcitability, seronegative autoimmune encephalitis

## Abstract

We report a 63‐year‐old Palestinian man with seronegative Morvan syndrome presenting with the complete clinical triad: continuous neuromyotonia with visible myokymia, severe insomnia consistent with agrypnia excitata phenotype (2–3 h/night; Insomnia Severity Index [ISI] 24/28, validated Arabic version), tachycardia (118 bpm), profuse diaphoresis, and 13‐kg weight loss. Electromyography (EMG) demonstrated characteristic myokymic (40–150 Hz) and neuromyotonic (> 150 Hz) discharges across 12 muscles without denervation. Brain MRI, CSF analysis, and comprehensive malignancy screening were unremarkable. Validated cell‐based assays confirmed seronegativity for CASPR2 and LGI1 in both serum and CSF. A phenotype‐supported diagnosis was pursued integrating the clinical triad, electrophysiological confirmation, and systematic exclusion of mimics. Combined immunotherapy (intravenous methylprednisolone, prednisone taper, azathioprine) and symptomatic treatment (carbamazepine, melatonin) resulted in marked clinical improvement at 6 months: Medical Research Council (MRC) sum score 48 → 58/60, creatine kinase (CK) 928 → 185 U/L, ISI 24 → 4, and sleep restored to 7–8 h/night, with no significant adverse effects. This case illustrates a phenotype‐supported diagnostic approach applicable in resource‐limited settings where first‐line biological therapies are unavailable.

## Key Clinical Message

Seronegative Morvan syndrome should remain a clinical consideration when neuromyotonia, severe insomnia consistent with agrypnia excitata, and dysautonomia are supported by characteristic electrophysiology and exclusion of mimics. Combined corticosteroid‐based immunotherapy achieved marked improvement in a resource‐limited setting.

## 1 Introduction

Morvan syndrome is a rare autoimmune neurological disorder (incidence < 1 per million) characterized by the clinical triad of peripheral nerve hyperexcitability (PNH; neuromyotonia/myokymia), severe insomnia consistent with agrypnia excitata, and dysautonomia [1]. It is linked to voltage‐gated potassium channel (VGKC) complex antibodies—particularly CASPR2 and LGI1—in a subset of patients [1, 2], but validated antibody testing identifies these in only 40%–60% of clinically consistent cases [2, 3] leaving a substantial seronegative proportion. Seronegativity may reflect T‐cell‐mediated mechanisms, antibodies targeting uncharacterized VGKC epitopes, or detection‐threshold limitations—each representing a hypothesis rather than an established mechanism [4, 5]. In resource‐limited settings, this creates profound diagnostic and therapeutic uncertainty. We present a rigorously documented seronegative case illustrating a phenotype‐supported diagnostic approach and the clinical outcomes of combined corticosteroid‐based immunotherapy and symptomatic treatment, in adherence to CARE guidelines [12].

## 2 Case Presentation

### 2.1 Clinical History and Timeline

A 63‐year‐old Palestinian man was referred to the neurology clinic in August 2024 with a 6‐month history of progressive muscle twitching, insomnia, and autonomic dysfunction. History included a 70‐pack‐year smoking history (cessation 5 years prior) and well‐controlled hypertension on amlodipine. No history of autoimmune disorder, malignancy, or neurological disease; family history unremarkable. The clinical timeline is detailed in Table 1.

**TABLE 1:** Clinical timeline: symptom evolution.

Month	Date	Clinical events
0	Feb 2024	Intermittent fasciculations, bilateral lower extremities
1	Mar 2024	Continuous fasciculations, upper extremities and trunk; nocturnal cramps
2	Apr 2024	Severe insomnia (7–8 h → 2–3 h/night); vivid disturbing dreams—consistent with agrypnia excitata phenotype
3	May 2024	Profuse diaphoresis; palpitations (HR 105–120 bpm); weight loss ~5 kg
4–5	Jun–Jul 2024	Proximal weakness (stairs, overhead lifting); total weight loss 13 kg
6	Aug 2024	Hospital presentation (69 kg from 82 kg); phenotype‐supported diagnosis; treatment initiated
6‐mo FU	Feb 2025	Marked improvement: MRC^a^ 58/60; CK^b^ 185 U/L; sleep 7–8 h/night; ISI^c^ 4/28; autonomic resolution

^a^ MRC: Medical Research Council sum score (bilateral shoulder abductors, elbow flexors, wrist extensors, hip flexors, knee extensors, ankle dorsiflexors; each 0–5; max 60).

^b^ CK: Creatine kinase (ref 30–200 U/L).

^c^ ISI: Insomnia Severity Index; validated Arabic‐language version; score ≥ 15 = severe insomnia.

### 2.2 Examination Findings

Vital signs: BP 138/82 mmHg (no orthostasis); HR 118 bpm; body mass index (BMI) 21.3 kg/m^2^; profuse palmoplantar and frontal diaphoresis. Cognitive assessment preserved (Mini‐Mental State Examination (MMSE) 29/30; Montreal Cognitive Assessment (MoCA) 27/30). Motor examination: visible continuous rippling in bilateral deltoids and vastus lateralis (marked), biceps and gastrocnemius (moderate). MRC sum score 48/60 (symmetric proximal weakness; distal strength preserved). Reflexes 2+ throughout; plantars flexor; sensation and coordination intact. The clinical picture—visible neuromyotonia, insomnia consistent with agrypnia excitata phenotype, and autonomic overactivity—constituted the complete characteristic triad.

## 3 Investigations

### 3.1 Electrophysiological Studies

**FIGURE 1:**
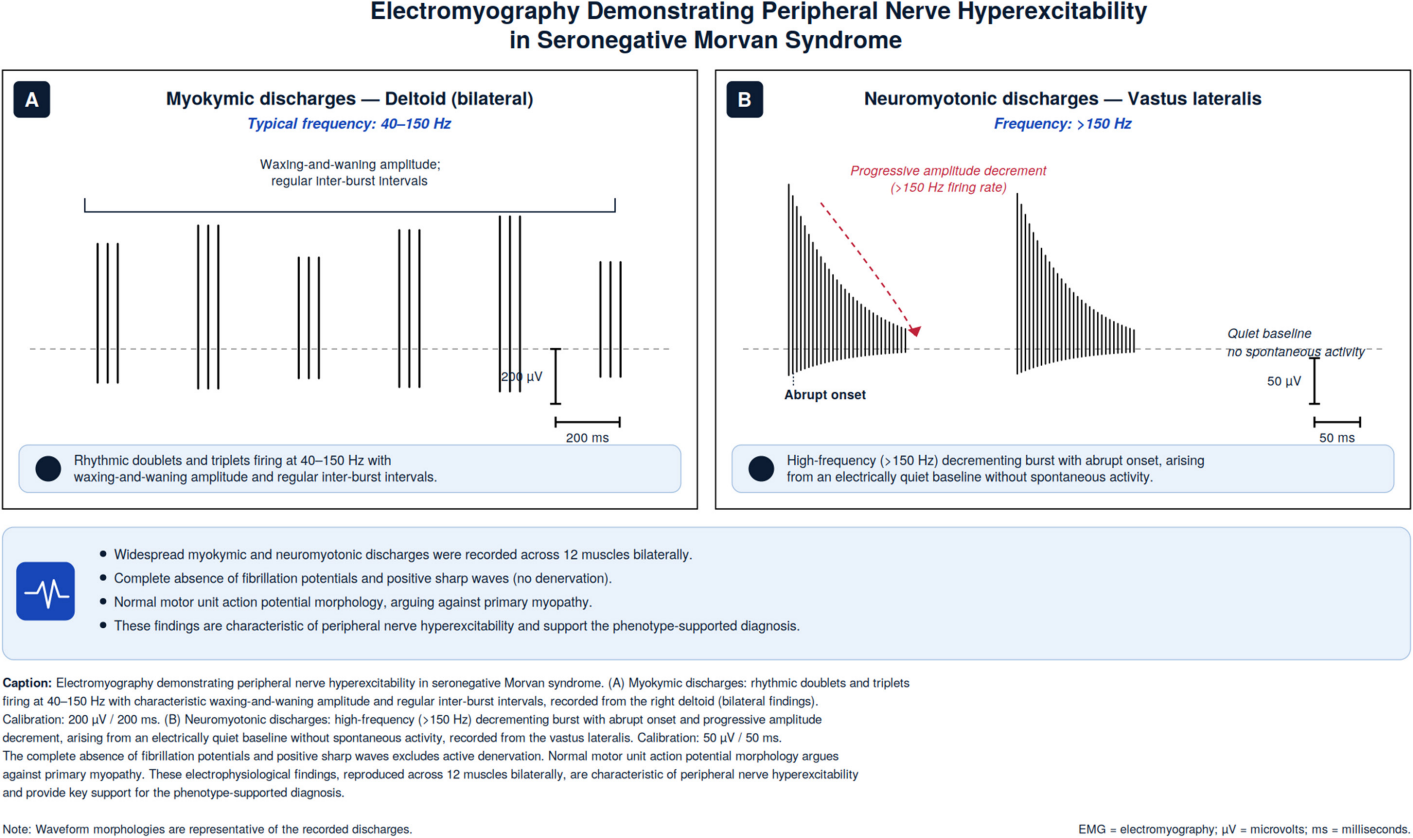
Electromyography demonstrating peripheral nerve hyperexcitability in seronegative Morvan syndrome. (A) Myokymic discharges: Rhythmic doublets and triplets firing at 40–150 Hz with waxing‐and‐waning amplitude and regular inter‐burst intervals. Calibration: 200 μV/200 ms. (B) Neuromyotonic discharges: High‐frequency decrementing bursts (> 150 Hz) with abrupt onset and progressive amplitude decrement, arising from a quiet baseline. Calibration: 50 μV/50 ms. Absence of fibrillation potentials excludes denervation; normal MUAP morphology excludes primary myopathy. These findings are characteristic of peripheral nerve hyperexcitability and are central to the phenotype‐supported diagnosis.

EMG demonstrated characteristic myokymic discharges (40–150 Hz) across 12 muscles and neuromyotonic discharges (> 150 Hz) in bilateral deltoid and vastus lateralis, with no fibrillation potentials or positive sharp waves, excluding active denervation. Normal motor unit action potential (MUAP) morphology excluded primary myopathy. Nerve conduction studies were normal. Detailed findings are summarized in Table 2; representative waveforms are illustrated in Figure 1.

**TABLE 2:** EMG results: peripheral nerve hyperexcitability documentation.

Muscle	Side	Myokymic discharges	MUAP^a^ characteristics
Deltoid	Bilateral	+++ Triplets, 70–120 Hz; neuromyotonic bursts > 150 Hz	Normal; mildly reduced recruitment
Biceps brachii	Bilateral	++ Doublets/triplets, 50–100 Hz	Normal; mildly reduced recruitment
First dorsal interosseous	Bilateral	+ Doublets, 40–70 Hz	Normal; normal recruitment
Vastus lateralis	Bilateral	+++ Triplets, 85–150 Hz; neuromyotonic bursts > 150 Hz	Normal; mildly reduced recruitment
Iliopsoas	Bilateral	++ Doublets/triplets, 65–110 Hz	Normal; mildly reduced recruitment
Tibialis anterior	Bilateral	+ Doublets, 45–80 Hz	Normal; normal recruitment
**Summary (12 muscles)**	All	Characteristic myokymic discharges in all 12 muscles; neuromyotonic bursts: bilateral deltoids and vastus lateralis; proximal > distal; no denervation	Normal MUAP morphology throughout; primary myopathy excluded

^a^ MUAP: Motor unit action potential. Grading: +++ = continuously present; ++ = frequently present; + = intermittently present.

### 3.2 Laboratory and Autoantibody Investigations

CK 928 U/L (4.6× upper limit of normal [ULN]); sodium 133 mmol/L; thyroid function normal (thyroid‐stimulating hormone [TSH] 2.4 mIU/L); inflammatory markers mildly elevated. Validated cell‐based assays (EUROIMMUN): CASPR2 and LGI1 negative (< 1:10) in serum and CSF; VGKC complex 45 pmol/L (below diagnostic threshold of 100 pmol/L—non‐specific; reported for completeness; not interpreted as supportive evidence) [15, 16]. NMDA/AMPA/GABA‐A/B, paraneoplastic (Hu, Yo, Ri, Ma2, GAD65), and connective tissue antibodies all negative. Thiopurine methyltransferase (TPMT)/nudix hydrolase 15 (NUDT15) genotyping was unavailable; azathioprine was commenced at a test dose (50 mg/day) with fortnightly complete blood count (CBC) and liver function tests (LFTs); no hematological adverse events occurred.

### 3.3 CSF, Neuroimaging, and Malignancy Screening

CSF: normal cell count, protein, glucose; oligoclonal bands negative; cultures and viral polymerase chain reaction (PCR) for herpes simplex virus (HSV) and varicella‐zoster virus (VZV) negative. Brain MRI (1.5 T, gadolinium): age‐appropriate white matter changes only; no limbic, temporal lobe, or hippocampal signal abnormality. Malignancy screening (given 20%–40%) Morvan–malignancy association [10, 11]: computed tomography (CT) chest/abdomen/pelvis, tumor markers (carcinoembryonic antigen [CEA], alpha‐fetoprotein [AFP], carbohydrate antigen 19‐9 [CA19‐9], prostate‐specific antigen [PSA]), upper endoscopy, colonoscopy, and testicular ultrasound—all negative.

## 4 Diagnostic Synthesis

### 4.1 Differential Diagnosis and Systematic Exclusion

Alternative conditions were systematically excluded, as detailed in Table 3. Key exclusions: motor neuron disease (no denervation on EMG, no upper motor neuron signs); primary myopathy (neurogenic EMG pattern, normal MUAP morphology); myasthenia gravis (no fatigable weakness, normal repetitive nerve stimulation (RNS)); limbic encephalitis (MMSE 29/30, normal temporal MRI, normal CSF); CNS infection (normal CSF, negative viral PCR); paraneoplastic syndrome (negative antibodies and malignancy screen); thyrotoxicosis (normal thyroid function).

**TABLE 3:** Differential diagnosis and systematic exclusion.

Condition	Key distinguishing features	Reason excluded
Motor neuron disease	Denervation on EMG; upper motor neuron signs	No fibrillation/positive sharp waves; no upper motor neuron (UMN) signs
Primary myopathy	Myopathic EMG; proximal weakness	Neurogenic (myokymic) EMG pattern; normal MUAP morphology
Myasthenia gravis/neuromuscular junction (NMJ)	Fatigable weakness; decremental RNS	No fatigable weakness; normal RNS
Limbic encephalitis	Cognitive dysfunction; temporal MRI changes; CSF pleocytosis	MMSE 29/30; normal MRI and CSF
CNS infection	Fever; CSF pleocytosis; positive PCR	Normal CSF; negative HSV/VZV PCR
Paraneoplastic syndrome	Underlying malignancy; paraneoplastic antibodies	Negative antibodies and malignancy screen
Thyrotoxicosis/metabolic	Abnormal thyroid function; metabolic disorder	Normal thyroid function tests (TFTs) (TSH 2.4 mIU/L); normal metabolic panel

### 4.2 Phenotype‐Supported Diagnostic Framework

**FIGURE 2:**
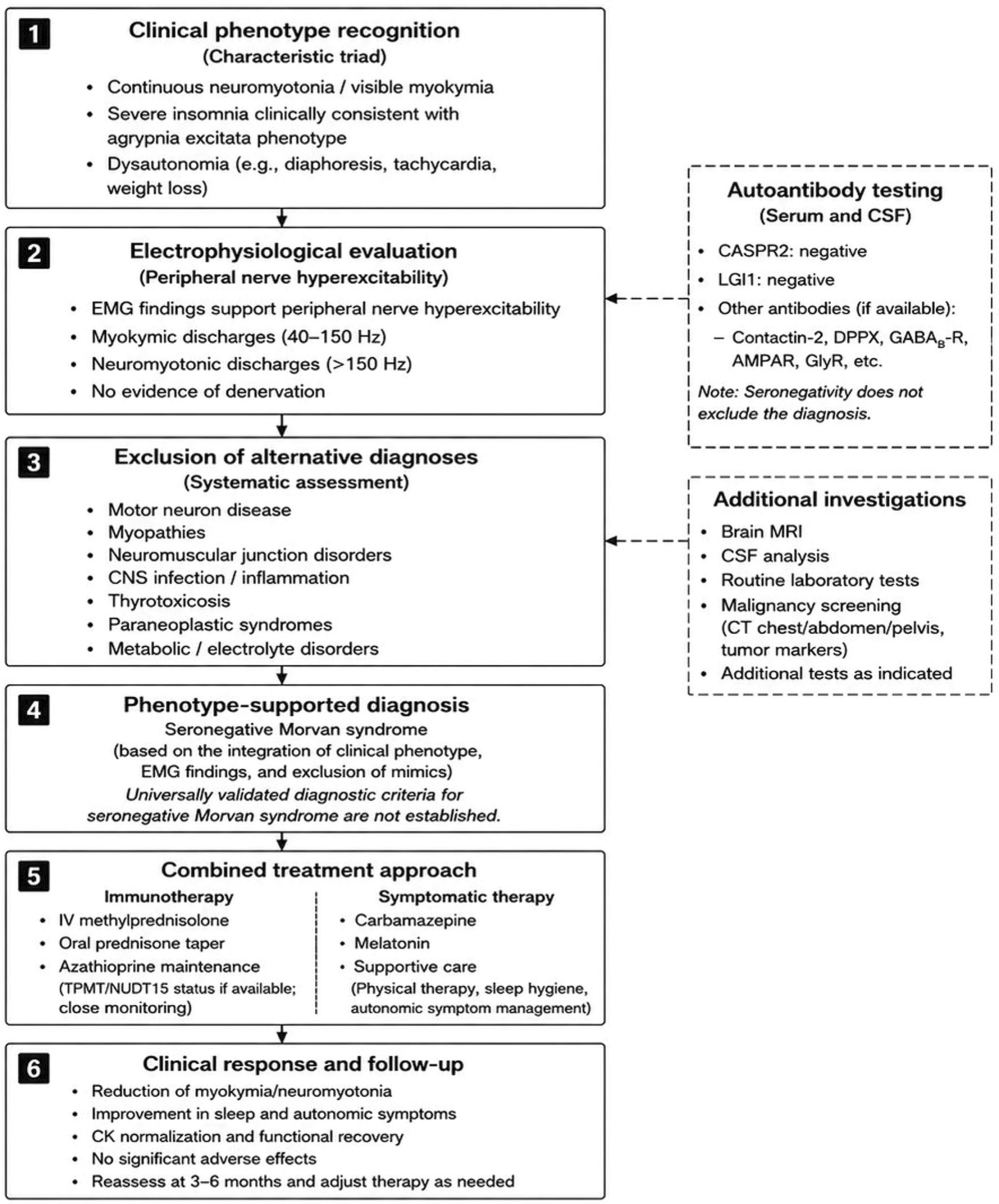
Phenotype‐supported diagnostic and therapeutic flowchart for seronegative Morvan syndrome. Universally validated diagnostic criteria for seronegative Morvan syndrome are not established; the preferred formulation is “phenotype‐supported diagnosis.”

Although universally validated diagnostic criteria for seronegative Morvan syndrome are not established, the diagnosis was supported—rather than definitively confirmed—by the complete clinical triad, characteristic EMG findings, and systematic mimic exclusion, following a modified Irani criteria‐based approach [6]. The stepwise pathway is summarized in Table 4 and illustrated in Figure 2.

**TABLE 4:** Phenotype‐supported diagnostic pathway.

Step	Evaluation	Findings in this case
1	Clinical triad (PNH, agrypnia excitata phenotype, dysautonomia)	All 3 present: neuromyotonia; sleep 2–3 h/night (ISI 24); tachycardia + diaphoresis
2	EMG confirmation	Characteristic myokymic/neuromyotonic discharges (12 muscles); no denervation; normal MUAP morphology
3	Autoantibody testing (serum and CSF)	CASPR2 and LGI1 negative; VGKC 45 pmol/L (below threshold; non‐specific)
4	Systematic mimic exclusion	All mimics excluded (Table 3)
5	Malignancy screening	Comprehensive screen negative; thymoma excluded
6	Combined immunotherapy + symptomatic treatment	Initiated; clinical response consistent with immune‐mediated pathophysiology

## 5 Treatment and Follow‐Up

### 5.1 Treatment Protocol and Rationale

Intravenous immunoglobulin (IVIG) and plasma exchange (PLEX) were inaccessible (estimated costs: IVIG $6000–10,000; PLEX > $10,000). Corticosteroid‐based immunotherapy with azathioprine was selected as a cost‐effective alternative (~$350 total for 6 months), consistent with published reports [8, 9]. These cost estimates reflect local pricing and healthcare resource availability at our institution and may not be generalisable to other settings. Three‐phase protocol: (1) IV methylprednisolone 1000 mg/day × 3 days, then oral prednisone 60 mg/day tapered by 10 mg every 2 weeks to 10 mg/day at 3 months; (2) azathioprine 50 mg/day test dose (Week 2) escalated to 150 mg/day (2.2 mg/kg) with CBC/LFT monitoring; (3) carbamazepine 200 mg twice daily escalated to 400 mg twice daily (Week 2), melatonin 5 mg at bedtime, and vitamin D 2000 IU/day.

### 5.2 Treatment Response and Outcome

Clinically meaningful improvement was observed from Week 1. The patient received concurrent immunotherapy and symptomatic agents; accordingly, the relative contribution of each component cannot be determined from this single‐patient observation. Quantitative responses are detailed in Table 5. At 6 months: MRC 48 → 58/60; CK normalized (928 → 185 U/L); ISI improved 24 → 4; sleep restored to 7–8 h/night; heart rate 72–85 bpm; weight regained 8 kg; myokymia barely detectable; patient returned to full work. No significant adverse effects were recorded.

**TABLE 5:** Clinical and biochemical response to combined treatment (6‐month trajectory).

Time	Regimen	MRC	CK (U/L)	Sleep (h)	ISI	Myo‐kymia	Adverse effects
Baseline	None	48	928	2–3	24	+++	—
Week 1	IV MP^a^ 1 g × 3 days; Pred^b^ 60 mg; CBZ^c^ 200 mg BD	49	—	4–5	22	++	Mild flushing (resolved)
Week 2	Pred^b^ 60 mg; AZA^d^ 50 mg; CBZ^c^ 400 mg BD	50	820	5	20	++	No hematological toxicity
Month 1	Pred^b^ 50 mg; AZA^d^ 150 mg; CBZ^c^ 400 mg BD	52	485	5–6	16	++	Mild nausea (AZA, self‐limiting)
Month 2	Pred^b^ 40 mg; AZA^d^ 150 mg; CBZ^c^ 400 mg BD	54	325	6	12	+	None
Month 3	Pred^b^ 30 mg; AZA^d^ 150 mg; CBZ^c^ 400 mg BD	56	245	6–7	8	+	None
Mo 4–5	Pred^b^ 20 → 10 mg; AZA^d^ 150 mg; CBZ^c^ 400 mg BD	57	210	7	6	+/−	None
Month 6	Pred^b^ 10 mg; AZA^d^ 150 mg; CBZ^c^ 400 mg BD	58	185	7–8	4	+/−	None
Δ Total	—	+10	−743	+5 h	−20	Near resolved	—

^a^ MP: Methylprednisolone.

^b^ Pred: Prednisone.

^c^ CBZ: Carbamazepine.

^d^ AZA: Azathioprine. BD: twice daily. Myokymia: +++ continuously visible; ++ frequently; + intermittently; +/− barely detectable. Note: Concurrent immunotherapy and symptomatic agents were administered; attribution to any single component is not possible.

### 5.3 Ongoing Management and Surveillance

Maintenance: prednisone 10 mg (taper at month 9; discontinue month 12); azathioprine 150 mg/day (minimum 18–24 months); carbamazepine 400 mg twice daily (taper month 9); monthly CBC, LFTs, and CK. Malignancy surveillance (local clinical decision—not a universal protocol): CT chest/abdomen/pelvis every 6 months for years 1–2, then annually for years 3–5; age‐appropriate cancer screening. Potential cumulative radiation exposure was discussed with the patient as part of shared decision‐making.

## 6 Discussion

### 6.1 Pathophysiologic Hypotheses and Diagnostic Approach

In seronegative Morvan syndrome, two mechanistic hypotheses may plausibly account for the phenotype: T‐cell‐mediated cytotoxicity targeting VGKC‐associated proteins and antibodies targeting uncharacterized VGKC epitopes (CNTN2, ADAM22, DPPX) below detection thresholds [15, 16]. The VGKC complex value of 45 pmol/L was below the accepted diagnostic threshold (100 pmol/L) and is not interpreted as supportive evidence; it is reported for completeness. Clinical improvement within the first week of high‐dose methylprednisolone is biologically compatible with reversible immune‐mediated ion‐channel dysfunction, though this inference remains hypothesis‐generating in the context of a single observation with concurrent symptomatic treatment. Published cohorts suggest that a substantial proportion of clinically consistent Morvan syndrome cases may remain seronegative, although reported frequencies vary across studies [2, 3]. Diagnostic support rested on: the complete characteristic triad; characteristic myokymic/neuromyotonic discharges across 12 muscles without denervation; comprehensive cell‐based assays in serum and CSF; and systematic mimic exclusion. Polysomnography was unavailable; sleep outcomes relied on the validated Arabic‐language ISI, which cannot objectively confirm agrypnia excitata—sleep disturbance is accordingly described as consistent with agrypnia excitata phenotype rather than objectively confirmed.

### 6.2 Comparison With Published Literature

Table 6 compares this case with the two largest published Morvan syndrome cohorts. The patient's demographic profile, complete triad, and seronegative status are consistent with published descriptions [3, 6]. Following combined immunotherapy and symptomatic treatment, the patient achieved marked clinical improvement (MRC 48 → 58/60; CK 928 → 185 U/L; ISI 24 → 4; sleep 2–3 → 7–8 h/night), which appears broadly consistent with the range of outcomes reported in published cohorts; however, direct comparisons are limited by differences in study design, antibody status, treatment regimens, and outcome assessment.

**TABLE 6:** Present case vs. major published Morvan syndrome cohorts.

Feature	Present case	Irani 2012 [6] (*n* = 29)	van Sonderen 2016 [3] (*n* = 140)
Age (years)	63	Median 62	Median 60
Sex	Male	90% male	88% male
Neuromyotonia	Continuous; visible myokymia	100%	84%
Agrypnia excitata phenotype	Severe (2–3 h; ISI 24)	90%	57%
Autonomic dysfunction	Tachycardia + diaphoresis	76%	52%
Seronegative	Yes (CASPR2/LGI1 −)	~31%	N/A (CASPR2+ cohort)
Associated malignancy	None detected	38%	21%
Treatment response	Marked (MRC +10; CK −743; ISI −20)	Good–excellent 69%	Complete/good 62%

### 6.3 Treatment in Resource‐Limited Settings and Malignancy Surveillance

In our local healthcare setting, the estimated direct treatment cost of the corticosteroid–azathioprine regimen was approximately $350 over 6 months, compared with estimated local costs of $6000–10,000 for IVIG and > $10,000 for PLEX [27]. These figures are context‐specific and may vary substantially across healthcare systems. Published reports support corticosteroid‐based regimens in seronegative cases [6, 8]. Escalation to IVIG, PLEX, or rituximab would be considered for rapid deterioration, steroid contraindications, or non‐response after 4–6 weeks. The 20%–40% tumor association necessitates comprehensive malignancy screening regardless of antibody status [10, 11]; whether seronegative cases carry equivalent risk remains uncertain. The surveillance plan reflects local clinical judgment and should be individualized with due consideration of cumulative radiation exposure and patient preferences.

### 6.4 Limitations

Acknowledged limitations include: (1) absence of polysomnography—sleep disturbance is described as consistent with agrypnia excitata phenotype rather than objectively confirmed; (2) unavailability of TPMT/NUDT15 genotyping (mitigated by low‐dose AZA initiation and close laboratory surveillance), human leukocyte antigen (HLA) typing, T‐cell assays, and ultra‐sensitive antibody testing (DPPX, IgLON5, CNTN2/ADAM22); (3) concurrent treatments preclude attribution of improvement to any single agent; (4) 6‐month follow‐up limits long‐term relapse assessment; (5) single‐centre experience with inherent case‐reporting methodological limitations.

## 7 Conclusions

A phenotype‐supported diagnosis of seronegative Morvan syndrome can be pursued using a systematic approach integrating the complete clinical triad, electrophysiological confirmation, and systematic exclusion of mimics—even without CASPR2/LGI1 antibody confirmation. Antibody negativity should not delay diagnostic consideration or treatment initiation when the characteristic phenotype is present. Combined corticosteroid‐based immunotherapy and symptomatic treatment resulted in marked clinical improvement at considerably lower cost than first‐line biological therapies, illustrating a clinically reasonable approach in resource‐limited settings. Structured malignancy surveillance remains essential regardless of antibody status.

## 8 Learning Points

- Antibody negativity should not exclude Morvan syndrome when the complete clinical triad, characteristic electrophysiology, and systematic exclusion of mimics are all present.
- EMG demonstrating characteristic myokymic and neuromyotonic discharges without denervation is central to supporting peripheral nerve hyperexcitability and distinguishing Morvan syndrome from motor neuron disease and myopathy.
- Severe sleep disturbance consistent with agrypnia excitata phenotype is a cardinal feature; polysomnography is the gold standard for objective characterization; validated insomnia scales provide only a subjective severity measure in its absence.
- Combined corticosteroid‐based immunotherapy and symptomatic treatment may achieve marked clinical improvement when IVIG and plasma exchange are inaccessible; relative contributions of individual agents cannot be determined from a single‐patient observation.

## Author Contributions

**Hatem Mousa Taha:** conceptualization, investigation, data curation, formal analysis, visualization, writing – review and editing, writing – original draft, supervision. **Khaled Hatem Taha:** investigation, data curation, writing – review and editing.

## Funding

The authors have nothing to report.

## Ethics Statement

This case report was conducted in accordance with the Declaration of Helsinki and CARE guidelines. Written informed consent was obtained from the patient. All identifying information has been removed.

## Conflicts of Interest

The authors declare no conflicts of interest.

## Data Availability

All relevant clinical data are included within the manuscript. Additional de‐identified data are available from the corresponding author upon reasonable request.
